# Impact of social determinants on opioid use disorder treatment engagement after release from jail: a case note content analysis

**DOI:** 10.1186/s13722-026-00666-2

**Published:** 2026-04-21

**Authors:** Justin S. Bell, Jodie M. Dewey, Dennis P. Watson, Carol Johnstone, John S. Palmer, Rachel Kurz, Christine E. Grella

**Affiliations:** 1 Lighthouse Institute, Chestnut Health Systems, 221 W Walton St, Chicago, IL 60610 USA; 2 Johnstone Consulting, LLC, Chicago, IL USA

**Keywords:** Incarceration, Substance-related disorders, Motivational interviewing

## Abstract

**Background:**

Despite strong evidence that sustained engagement in OUD treatment reduces overdose risk, individuals leaving jail face substantial barriers to initiating and maintaining care. Motivational interventions such as motivational interviewing (MI) are commonly used during this reentry period to enhance engagement. While MI effectively targets internal readiness to change, it is less clear how such approaches operate in the presence of persistent structural barriers. This study explores how social determinants of health (SDOH) factors shaped participant engagement during a motivational intervention following release from jail.

**Methods:**

We conducted a qualitative content analysis of 500 clinical case notes generated by personnel facilitating linkage to treatment for participants (*n* = 155) with OUD and recent criminal legal system involvement. Notes were generated during a clinical trial (NCT04365920, Registered 2019-12-19) of Recovery Management Checkups (RMC); RMC is an evidence-based intervention that uses ongoing “check-up” meetings grounded in MI to link individuals to substance use treatment and support their treatment retention and recovery, or re-link them to treatment as needed. Case notes were coded according to a framework adapted from the Centers for Disease Control and Prevention’s SDOH domains, and categorized as either facilitators or barriers to participants’ treatment engagement.

**Results:**

Findings highlighted economic stability as the most frequently cited barrier to treatment engagement, particularly housing instability, unemployment, and lack of transportation. Other barriers included poor physical health, legal constraints, and negative social influences. Facilitators included support from family and community, healthcare access, mutual aid involvement, and religious engagement.

**Conclusions:**

Results revealed how social and structural barriers impede treatment engagement within the context of a motivational-based linkage intervention. These findings underscore the need for integrated approaches that acknowledge both motivational readiness and the structural realities of participants' lives. Interventions may benefit from pairing motivational strategies with practical supports (e.g., transportation, housing navigation, harm-reduction planning). Given the challenges intervention staff face when attempting to help individuals in low-resource environments, implications for supporting personnel delivering motivational strategies are discussed.

Engagement in treatment for opioid use disorders (OUD) is strongly influenced by social determinants of health (SDOH), the environmental conditions in which people are born, live, work, and age [1–4]. Prior research consistently demonstrates that barriers such as homelessness, unemployment, poverty, lack of insurance, and racialized stigma are associated with reduced treatment participation and higher dropout risk [5–9]. These challenges are particularly salient among individuals involved in the criminal legal system (CLS), who experience disproportionately high rates of housing instability, uninsurance, and unmet behavioral health needs [10–13].

Although the role of SDOH in shaping treatment access and participation is well established, less attention has been paid to how these factors interact with evidence-based interventions and shape their effectiveness for populations facing structural barriers after incarceration [1, 14, 15]. Participation in OUD treatments during reentry, especially with medications for opioid use disorder (MOUD), have been found to reduce overdose risk and associated opioid-related mortality [16]. Therefore, understanding how SDOH shape access to and participation in OUD services following incarceration is critical for tailoring interventions to meet the needs of CLS-involved individuals and improving post-release outcomes [17].

## Motivational interventions

Historically, models of substance use disorder (SUD) intervention – particularly with justice-involved populations – relied on confrontational approaches that focused on “breaking down” an individual’s defenses (i.e., “denial”) and social identity as an “addict” [18, 19]. Over the past several decades, engagement strategies have shifted towards motivational approaches that emphasize empathy, autonomy, and collaboration [20–22]. *Motivational Interviewing* (MI), an evidence-based, client-centered method designed to strengthen intrinsic motivation for change, has become widely institutionalized within SUD programming [23]. Its use with justice-involved populations is especially notable [24]. MI has achieved near universal implementation in some US justice settings (e.g., 97% of juvenile probation departments in Pennsylvania; [25]) and is a common requirement of probation and parole officer training, often used to facilitate referrals to SUD treatment [26, 27].

Evidence suggests that MI-based interventions can improve treatment attendance and reduce dropout among justice-involved individuals compared to non-MI approaches [28, 29]. However, although MI is designed to enhance motivation and confidence for behavior change, successful follow-through depends on access to material and social resources [30, 31]. Individuals must be able to secure transportation, stable housing, healthcare, and other supports to meet treatment obligations. When these structural barriers impede attendance, individuals may be labeled “resistant” or “non-compliant,” potentially undermining trust and practitioner rapport [31].

Although MI emphasizes a collaborative process in which practitioners and participants can identify and problem-solve barriers together, recent commentary notes that evaluations of MI have rarely examined how SDOH shape its effectiveness [32]. For example, a participant may leave an MI session motivated to attend treatment but is realistically constrained by other priorities in their life. Indeed, studies note that women with CLS-involvement may prioritize family pressures, caring for children, and access to employment over the intended behavioral changes of SUD interventions [28, 33, 34]. Understanding how SDOH-related barriers interact with MI is therefore critical for tailoring engagement strategies among individuals with justice-involvement.

### Recovery management check-ups

Recovery Management Checkups (RMC) is a motivational, evidence-based intervention that aims to link individuals to SUD treatment and recovery services by providing ongoing recovery support through a person-centered public health model of chronic disease management [35–42]. Drawing heavily on use of MI, RMC aims to enhance motivation for attending treatment and recovery services. Across more than 25 years of research, including multiple RCTs with participants from various settings, participants receiving RMC return to treatment sooner, remain engaged longer, accumulate more treatment days, and report fewer days of substance use and related risk behaviors compared to controls [35–42].

Despite evidence of robust outcomes, many RMC participants still struggle to enter and sustain SUD treatment participation. Based on the investigators’ observations, a cursory review of case notes from the intervention’s linkage meetings, and debriefs with linkage managers, we determined that a systematic review of case notes may yield insights into how SDOH impacts treatment participation. Moreover, the longitudinal nature of the RMC intervention, delivered quarterly (or more frequently) for up to 24 months, affords multiple observation points and in-depth engagement with study participants.

### Current study

Case note documentation from RMC provides an opportunity to identify how SDOH influences treatment engagement during a motivational intervention. In situations where validated measures of SDOH are not used, case notes offer a rich narrative source that can capture the contextual and nuanced aspects of individuals’ social environments. Studies have shown that providers across disciplines frequently rely on narrative notes to document social needs, and recent research has successfully extracted meaningful SDOH data from these unstructured texts [43, 44].

The current study is a content analysis of case notes collected as part of a clinical trial of RMC delivered to individuals exiting jail [45]. We analyzed practitioner documentation to identify how SDOH shaped OUD treatment engagement and recovery following release from jail. Consistent with the goals and demonstrated outcomes of RMC, “engagement” is defined as enrollment and retention in substance use treatment and participation in recovery-oriented activities. Specifically, we addressed two research questions: (1) Which SDOH factors emerged as facilitators of engagement in OUD treatment and recovery? and (2) Which SDOH factors functioned as barriers to OUD treatment engagement and recovery among justice-involved participants receiving RMC? We further consider how these findings can inform the design and delivery of motivational interventions that are better aligned with the structural realities faced by individuals navigating post-incarceration re-.

## Methods

To examine individual- and systemic-level barriers and facilitators to treatment engagement and recovery, a content analysis was conducted of participant case notes generated during a randomized controlled trial of the RMC intervention conducted between 2021 and 2024 [45]. RMC was delivered to individuals with OUD following their release from one of six county jails (five in Illinois, one in Indiana). The parent trial was part of the National Institute on Drug Abuse-sponsored Justice Community Opioid Innovation Network (JCOIN) initiative and was approved by the [IRB #FWA00000497] in accordance with the Declaration of Helsinki and registered at ClinicalTrials.gov [NCT04365920; Registered 2019-12-19]. All participants provided written informed consent to participate in this study.

RMC is an ongoing, motivational interviewing (MI)-based intervention designed to support treatment linkage and retention and to support sustain recovery through structured “check-up” meetings over time. During these meetings, the RMC intervention staff – who are called *linkage managers* – use MI strategies to assess treatment motivation and readiness, explore barriers, and facilitate linkage to substance use treatment and recovery services; in successive check-ups they provide ongoing support for treatment retention and sustained recovery, or treatment re-linkage as needed. Following each meeting, linkage managers document participant-reported challenges, goals, and resource needs in structured case notes.

### Case notes

As part of their routine responsibilities, linkage managers documented each RMC meeting with study participants through brief clinical case notes in a centralized database that tracked all RMC encounters. These notes served as a tool for internal communication, case tracking, and continuity of care, capturing observations about participant progress, expressed needs, perceived barriers, and treatment-related decisions. Although linkage managers were not given formal instructions on how to structure their notes, they were encouraged to maintain ongoing documentation and to record key details immediately following each RMC session. Notes varied in length and content, reflecting the clinical judgment and style of each linkage manager.

### Content analysis

We employed a directed content analysis approach [46], using the Centers for Disease Control and Prevention’s (CDC) SDOH framework as an organizing structure (5). The initial coding framework included the five CDC domains: economic stability; education access and quality; healthcare access and quality; neighborhood and built environment; and social and community context. Codes were applied to identify segments of text describing factors that facilitated or hindered participants’ engagement in substance use treatment and recovery resources within each domain.

Case notes were sorted randomly and selected using Microsoft Excel from the full database of 2,118 RMC meeting notes generated for 305 study participants enrolled in the parent study who were randomly assigned to receive RMC (out of the total sample of 455). Linkage Managers documented all interactions with study participants in the database, i.e., date, meeting duration, participant’s current substance use and treatment status, treatment or other recovery services referred or scheduled, and an open-ended field for “case notes.” The final analytic sample included 500 case notes representing 155 unique study participants that were generated from seven linkage managers that worked on the study. Case notes were sampled such that 61.3% (*n* = 95) of study participants contributed more than one note, with an average of 2.79 case notes contributed per participant. Linkage managers contributed an average of 71.43 case notes each. The proportion of case notes contributed by individual linkage managers ranged from 1% to 35%. Variability was due, in part, to the duration of time individual linkage managers worked on the project.

Two raters (JB and JD) first familiarized themselves with the dataset by independently piloting the preliminary codebook on a subset of notes. Raters met regularly to refine operational definitions and ensure consistent application of codes. During this phase, we identified justice-specific structural influences that were not adequately captured by the original CDC framework and therefore added a sixth domain, “Policy and Institutional Context.”

Interrater reliability was assessed using Cohen’s kappa, with a target threshold of ≥ 0.70. Where reliability fell below this threshold, definitions were clarified and distinctions between codes were refined. Once the codebook was finalized and reliability consistently met the predetermined threshold across pilot rounds, the remaining case notes were independently coded. Disagreements were resolved through discussion with two additional reviewers (DW and CG). We also reviewed the proportion of each code attributed to each individual to prevent findings from being unduly driven by a small number of high-volume contributors, consistent with qualitative analytic approaches that emphasize within- and across-case comparison [47, 48]. Following coding, codes were summarized into higher-order facilitator and barrier categories within each domain. Only categories documented across notes from at least two different linkage managers were retained. A form of *member checking* was employed to increase the validity of our findings [49, 50]. This entailed sharing drafts of our results with an RMC linkage manager and supervisor (Authors JP and RK) for feedback on the interpretation of the notes and to add additional context where it was needed.

## Results

### Study sample

The present analysis focused on case notes from a sub-sample of 155 study participants in the parent JCOIN study. Participants in this sub-sample had a mean age of 46.3 years (*SD* = 11.3), and the majority were male (81.9%) and African American (67.7%), with 15.5% identifying as Hispanic, 8.4% as White, and the remainder as multiracial or other backgrounds. Consistent with study inclusion criteria, all reported opioid use in the 90 days prior to their most recent incarceration (prior to study entry), with over two-thirds reporting fentanyl use during that period and approximately one-third reporting an overdose.

This sub-sample of study participants closely resembled the socio-demographics of the larger parent study sample from which it was drawn, as well as displayed similar patterns of fentanyl use, overdose, and MOUD usage prior to study entry. Two thirds had spent less than two weeks in jail, and many had charges or court dates pending. Close to all (88%) met criteria for “severe” SUD at treatment entry and a majority (69%) had prior treatment with MOUD.

### Analysis results

**Fig. 1:**
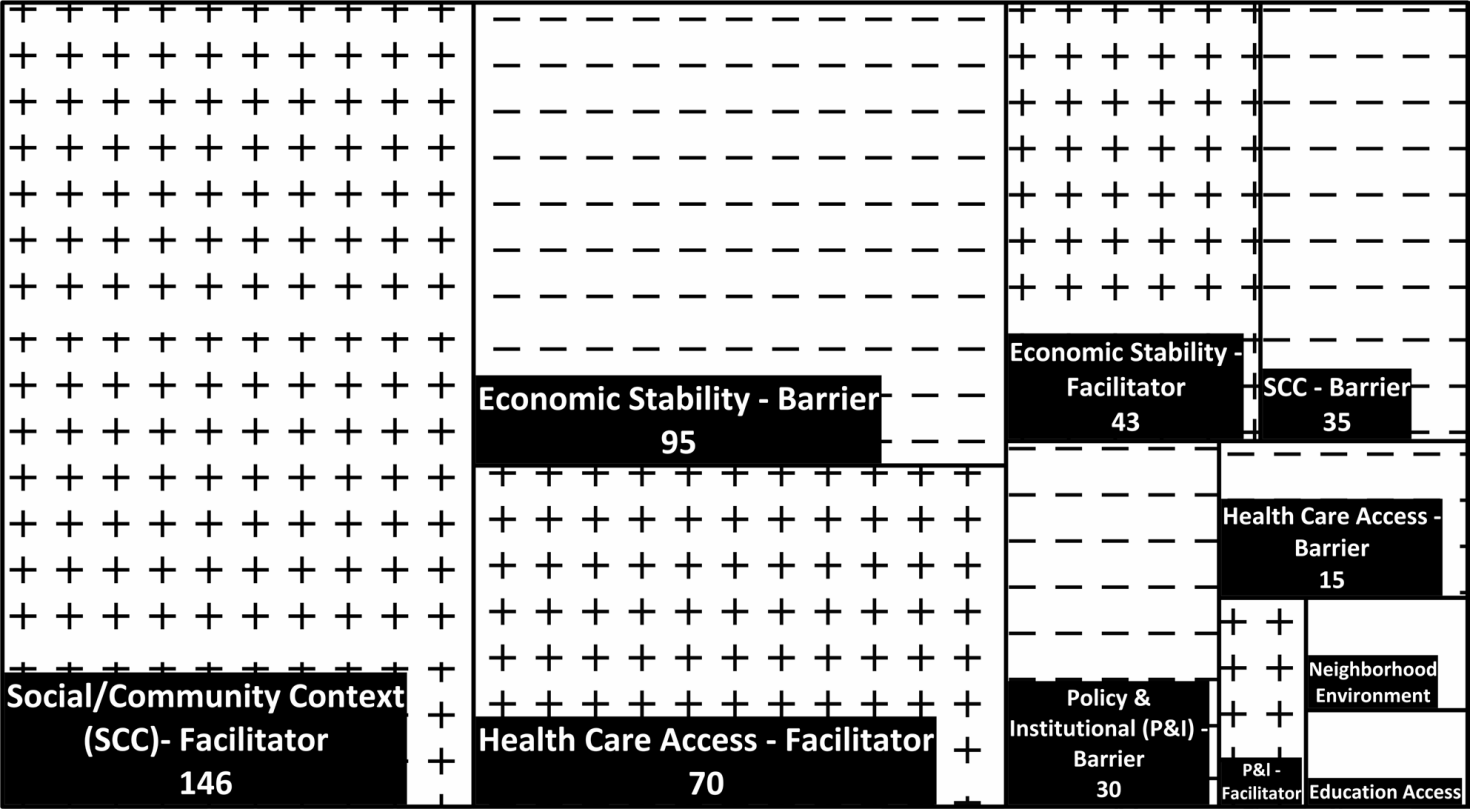
SDOH domains by frequency in case notes. *Note.* Facilitators to OUD treatment and recovery engagement are marked with pluses and barriers are marked with minuses. Numbers indicate case notes identified with these domains. Two domains were collapsed into both barriers and facilitators (*Neighborhood and Built Environment*,* Education Access;* marked in white). Individual case notes could be coded under multiple domains

Results present findings by domain, organized according to our SDOH framework and divided into facilitators and barriers: economic stability, social and community context, healthcare access and quality, policy and institutional context, educational access, and neighborhood and built environment. The overall distribution of notes coded by categories and sub-categories for barriers and facilitators is shown in Fig. 1. As seen, facilitators predominated, with social/community context, health care access, and economic stability most predominant. Among barriers, economic stability, social/community context, and policy–institutional factors predominated.

### SDOH facilitators

#### Social and community context facilitators

Across analyzed case notes, 29.2% (*n* = 146 of 500) referenced social and community factors that facilitated participants’ motivation to engage with treatment. These facilitators often involved meaningful relationships or sources of support embedded within participants’ personal and community environments. Three primary facilitators emerged: *Family*, *Mutual Aid*, and *Other Sources of Social Support*.

##### Family

Family served as both an internal and external motivator to engage with treatment. As an internal motivation, participants desired to become better family members and reunite with lost connections. Case notes described that because of their drug use, participants isolated themselves from family and missed key moments in their family member’s lives. These issues were reflected in expressed desires to become a better parent for their children, or a better grandparent. Participants were described as wanting to be more honest and devote more of their time to family. As an external motivation, family served as an important support network for many participants. Family members who explicitly supported their recovery could be counted on to motivate them during challenging times and sometimes encouraged them to return to treatment when they had disconnected. One note mentioned, “She still reaches out to her mom for support, mom is her big support system, since mom is also in recovery. [Participant] stays close to friends and family who are sober” (ID 2555, 2nd Monthly Meeting, Not in Treatment, 29 Years, Non-White Hispanic, Female).

##### Mutual aid support

Participation in recovery support meetings or mutual aid groups in the community also facilitated treatment engagement. “Meetings”, as they were typically referred to in the case notes, were mentioned as giving some participants a sense of routine, as well as a new support network. Participants often mentioned support from a “sponsor”, a member of a 12-step group chosen by the individual to provide guidance and accountability in recovery. Service providers often provided a setting to find and attend meetings. As a case note exemplified, “[Participant] also feels better about his [sobriety] and keeps busy going to meetings” (ID 2696, 1st Monthly, Not in Treatment, 57 Years, African American, Male).

##### Other sources of social support

Friends and religious practices also facilitated recovery and engagement in RMC. Positive, non-using social connections provided some participants with a source of motivation and replaced members of their social network who had once influenced them to use substances. Supportive romantic partners and other connections provided encouragement, as well as instrumental support, with one participant mentioning a friend supplying housing during a difficult time. Religion and spirituality also served as a source of support. Some participants mentioned attending religious services (i.e., “church”) as a fundamental source of support, or engaging in spiritual practices (i.e., “prayer”), as a note mentioned, “Has not used since his scary OD 10/19, is feeling better now about himself, recovery and has started attending church regularly” (ID 1457, 19th Monthly Meeting, Not in Treatment, 56 Years, African American, Male).

#### Health care access and quality facilitators

Approximately 14% of case notes documented factors that facilitated access to health care resources, which were primarily defined as treatment and recovery resources in this context. Two primary facilitators emerged: *Insurance and Identification* and *Resolving Health Concerns.*

##### Insurance and identification

Access to insurance and identification documents widened opportunities for health care resources. These assets help ensure quick linkage to services, helping to aid in sustained engagement, as exemplified by this note, “[Participant] has an ID and Aetna better health for insurance” (ID 1451, Baseline Meeting, Linking to Treatment, 54 Years, African American, Male).

##### Resolving health concerns (other than substance use)

Participants frequently indicated that addressing and managing their medical concerns increased their likelihood of engaging consistently with treatment. Many expressed that once their physical health was stabilized, they felt more capable of participating in other areas of care. Feeling healthier also reduces stress and improves one’s energy and attitude, something perceived as helping to increase treatment engagement and recovery. Addressing health conditions had an immediate motivational effect for participants, who could now focus on treatment, as a note exemplified, “[Participant] finally got his leg checked out. He is on antibiotics and ready to get back into the program” (ID 2865, 5th Monthly Meeting, Linking to MOUD Treatment, 58 Years, African American, Male).

#### Economic stability facilitators

Approximately 9% of analyzed case notes (*n* = 43) discussed factors related to economic stability as a facilitator of treatment engagement. Facilitators included *Employment & School*,* Housing Stability*, and *Access to Transportation*.

##### Employment & school

Case notes highlighted the significance of employment in enhancing both financial and personal well-being. Gaining and maintaining employment was not only a means of achieving financial stability, but also a marker of personal progress. For many RMC participants, securing a job marked a significant milestone in their recovery. Stable employment contributed to an individual’s overall sense of well-being and was frequently mentioned alongside other accomplishments or aspirations for self-improvement. Additionally, regular work provided structure and a constructive focus, helping individuals stay engaged and avoid potentially harmful behaviors. As one note described, “[She] has had an apartment for the last couple of months and now has a job and making meetings. [She] is doing well!” (ID 2156, 4th Quarterly Meeting, In MOUD Treatment, 35 Years, Caucasian, Female).

##### Housing stability

The case notes also underscored the critical importance of stable housing for participants. Housing served not only as a necessity but also as a symbol of personal growth and progress. Notes reflected that participants frequently associated stable living arrangements with a sense of safety, personal achievement, and a supportive foundation for maintaining treatment and sustaining recovery. Having a secure place to live was seen as essential to their ability to remain substance-free and focus on long-term goals. The positive effects were described in one note, “He moved into his new apartment, he feels great being clean and sober, he has quality housing and peace” (ID 2696, 7th Monthly Meeting, Not in Treatment, 58 Years, African American, Male).

##### Transportation access and support

Case notes identified that several participants viewed access to reliable transportation as a necessity to engage with treatment. Many relied on public transit to reach treatment services and expressed appreciation for the support provided through insurance-covered bus passes. This assistance was viewed as instrumental in maintaining consistent attendance and participation in care. The challenges of lacking a note were described in a note, “[Participant] has some issues w/ transportation. [Participant] agrees to try to come down to [study site] to get a bus card to ease the burden” (ID 1227, 19th Monthly Meeting, Not in Treatment, 54 Years, Non-White Hispanic, Male).

#### Policy & institutional context facilitators

Approximately 1% (*n* = 7) of the notes analyzed discussed that the CLS *Facilitated Treatment* and *Used Rewards and Punishments* to motivate participants to go to or stay in treatment.

##### CLS facilitated treatment

Case notes indicate that for several participants, involvement in the court process, particularly while on probation, served as a critical link to accessing and remaining in substance use treatment. As one note mentioned, “She is on probation and was told they would be sending her to inpatient” (ID 2555, 1st Monthly Meeting, Not in Treatment, 38 Years, Non-White Hispanic, Female).

##### CLS use of rewards and punishments

Additional case notes show the role the CLS system played in encouraging participants to enter treatment by either increasing or reducing one’s time in jail or on probation.

Also mentioned that he went to court and the judge lowered his sentence from 13yrs to 4yrs, he was so happy, but they want him to go to Inpatient 28-30 day program, referred [participant] to Gateway program, gave him # to get screened. [Participant] wants to continue to have a sober life and let the judge know he is serious about his sobriety. (ID 1681, 14th Monthly Meeting, Not in Treatment, 54 Years, Non-White Hispanic, Male)

In one case, a participant shared jail would be waiting if he continued to use substances, “He didn’t want to go to jail and said he made the changes because judge told him if he dropped dirty again, that was it for him” (ID 2242, 10th Monthly Meeting, In MOUD Treatment, 60 Years, Non-White Hispanic, Male).

### SDOH barriers

#### Economic stability barriers

Approximately 19% (*n* = 95) of the notes cited economic challenges as key factors undermining participants’ ability to access and remain in care. We identified a range of interconnected barriers in case notes that often compounded one another: *Housing Instability*,* Employment-Related Conflicts/Absence of Job Opportunities*,* Lack of Transportation*,* and Communication Difficulties*.

##### Housing instability

Case notes indicate that linkage managers frequently observed housing instability as a primary barrier to treatment engagement. Many participants expressed that they could not realistically consider entering treatment until they had secured stable housing. For some, the absence of housing, or the stress associated with unstable living conditions, exacerbated their substance use. Case notes reported that housing stability and security were essential prerequisites for engaging meaningfully in the recovery process, “[Participant] wants to secure housing before he gets into a program” (ID 1177, Baseline Meeting, Not in Treatment, 46 Years, African American, Male).

Those with precarious housing desired to focus on retaining the housing they had or find more stable housing, which for some was more than a personal desire but could potentially have legal consequences. As one note described, “He is currently going through an eviction process but missed his court date so he is on the verge of being homeless” (ID 2165, 5th Quarterly Meeting, Not in Treatment, 34 Years, African American, Male).

##### Employment-related conflicts/absence of job opportunities

Employment status also significantly influenced participants’ willingness and ability to engage in treatment. Employed participants were often recorded as expressing reluctance to enter treatment out of fear of jeopardizing their jobs. Similarly, unemployed participants prioritized seeking work over entering treatment, viewing employment as a foundational step toward stability. For these individuals, securing a job was seen as a pathway to obtaining housing, an essential condition they believed would reduce their substance use, increase their productivity, and help them regain control over their lives. As one note mentioned, “…he is tired of the problems caused by his drug use and would like to get a job” (ID 998, 2nd Quarterly Meeting, Linking to MOUD Treatment, 48 Years, African American, Male). For two participants unable to secure employment, selling drugs tethered them to the streets, as described by this note, “[Participant] *is ‘in the grips’*,* selling*” (ID 2556, 5th Monthly Meeting, Not in Treatment, 38 Years, African American, Male). A linkage manager who worked with this particular participant (ID 2556) shared that his home was condemned as it was identified as a site for drug sales. While economic conditions forced the participant into drug sales, he then was put in a tougher spot by losing his housing.

##### Lack of transportation

Case notes recorded that most participants relied on public transportation to access the services needed, including treatment. The time and money required to move around the city could be daunting and although bus cards were available for some, accessing them was equally difficult. As one note described, “…his biggest barrier now is transportation, which we can arrange this for him” (ID 1438, 1st Monthly Meeting, In MOUD Treatment).

##### Lack of phone

Two case notes referenced the disruption in participants’ lives because they had recently lost or had their phone stolen. Losing a phone made communication and follow-up with linkage managers difficult and led to delayed treatment support. As a note mentioned, “Lost her phone and all her belongings, staying w/ friends on the streets or when her friend lets her stay at her home. Mentioned that getting clean has been on her mind” (ID 3015, 1st Quarterly Meeting, Not in Treatment, 27 Years, Caucasian, Female).

#### Social and community context barriers

Across analyzed case notes, 7% (*n* = 35) referenced social and community-related barriers that hindered participants’ treatment engagement. These barriers often stemmed from interpersonal challenges or life responsibilities that conflicted with recovery goals. Three primary barriers emerged: *Negative Social Influences*,* Caretaking Responsibilities*, and *Challenging Life Situations.*

##### Negative social influences

Relationships could hinder recovery and engagement in treatment in several ways. Case notes described negative social influence from friends or other connections who continued to use substances. Case notes recorded that these participants described their problematic substance use was limited to when they were in contact, and desired to cut off these connections to focus on recovery. Participants shared how difficult it can be to sever these ties, particularly when such relationships were rooted in long-standing community bonds (or family) and participants lacked exposure to alternative, recovery-supportive networks. Two female participants described current or ex-partners that made them feel unsafe due to violent domestic altercations. Ultimately, case notes described that participants felt they could not fully engage with treatment or focus on their recovery when surrounded by people who distracted them from their recovery goals. As one note described,

He only uses cocaine but states he only uses when he is around certain friends. Right now he is out of town w/ his sister and hasn’t used. He knows he needs to stay away from them because he [wants] to live a better life and he is only using to please them. (ID 2636, 1st Monthly Meeting, Not in Treatment, African American, Male)

##### Caretaking responsibilities

Responsibilities as a caretaker for family members also served as a barrier to treatment. Several participants focused on their responsibility to care for their spouses and children. Female participants reported that they would not be able to engage with treatment as they had infants to care for or were soon to give birth. In one instance, a participant was documented as prematurely ending an RMC meeting because her children were ill. Others recounted being caretakers for older adult family members, who were often impaired. Again, these participants reported they could not attend treatment because there was no one else to care for their elderly family member as exemplified by a case note, “Currently still lives w/ his 81 year old auntie. Doesn’t have interest in going to TX at the moment, stated ‘when she moves I can move’” (ID 2885, 1st Monthly Meeting, Not in Treatment, 50 Years, African American, Male).

##### Challenging life situations

A smaller subset of participants were documented as experiencing challenging life situations that contributed to their substance use and unwillingness to engage with treatment. These included experiencing grief after the loss of a loved one or a personal relationship, as a note detailed, “[Participant] relapsed and starting using Heroin due to her recent heartbreak” (ID 1756, 19th Monthly Meeting, Attending MOUD treatment, 56 Years, African American, Female).

#### Policy & institutional context barriers

The most significant justice-related barriers reported in case notes (6%; *n* = 30) included disruptions due to *CLS Processes* and *Confinement*.

##### CLS processes

Participants pointed out the requirements of the CLS system as reasons for delaying treatment. Impending court dates, meetings with probation officers, and other conditions of community supervision were perceived as interruptions to treatment, as one case note described, “[Participant] continues to not want to commit to treatment. Has a court date in a month and will wait until then to decide” (ID 1161, 19th Monthly Meeting, Not in Treatment, 46 Years, Non-White Hispanic, Male).

Court dates created stress as participants were unaware of what could happen to them when they went in front of the judge, making plans for treatment difficult, as a note detailed, “[Participant] feels a little worried about going to court on [date] because he doesn’t know if the judge will see his accomplishments and take them into consideration” (ID 1681, 14th Monthly Meeting, Not in Treatment, Hispanic [Race Not Answered], Male).

Due to the unpredictability of court processes, many participants found it more feasible to fulfill court requirements prior to engaging in treatment. This was particularly true for those under community supervision, where movement restrictions frequently limited their ability to leave their residence to access services, as a note mentioned, “Still waiting to get approval from [parole officer] for movement that aligns with clinic dosing hours” (ID 1090, 1st Monthly Meeting, Linking to MOUD Treatment, 51 Years, African American, Male).

##### Confinement

Whether participants are sentenced to or arrested and held in correctional settings, periods of confinement disrupted and delayed treatment. Without access to treatment while confined, the time incarcerated can be challenging for participants engaged in treatment, as a note mentioned, “Got arrested and did 2 weeks in jail on an old warrant, still staying focused and clean” (ID 2136, 14th Monthly Meeting, Not in Treatment, 55 Years, African American, Male).

#### Health care access and quality barriers

Approximately 3% of case notes cited *Poor Physical Health* as the key barrier to treatment and recovery among participants.

##### Poor physical health

The most frequently cited health-related barrier to seeking treatment was participants’ ongoing physical health problems. Participants reported experiencing chronic medical conditions, as well as hospitalizations resulting from surgeries and accidents. These health challenges often took priority, with many participants expressing the need to resolve or stabilize their medical concerns before they felt able to fully commit to treatment, as a note detailed, “He also has to have hip surgery and wants to get his life back” (ID 2434, Monthly Meeting, In MOUD Treatment, 61 Years, African American, Male).

Others found that their chronic health conditions made it impossible to get to the clinic to receive treatment.

[Participant] would like to go back to methadone program but cannot go to [the clinic]. It’s too far for her to be walking and her legs swell up. Dr suggested she stay off of them and try not to walk much. (ID 2847, 1st Monthly Meeting, Linking to MOUD Treatment, 69 Years, African American, Female)

### Less common SDOH factors

#### Educational access

Approximately 1% (*n* = 6) of case notes discussed education-related barriers or facilitators. However, no single theme met criteria for inclusion. Of the 6 participants who discussed education, one participant indicated that he wanted to go back to school while the remaining five were enrolled in college or a training program. Training programs included a peer support specialist certification and a non-violence program, as one note detailed, “Starting school and working as a recovery coach” (ID 1322, 6th Quarterly Meeting, Not in Treatment, 31 Years, Non-White Hispanic, Male).

#### Neighborhood and built environment

Approximately 1% (*n* = 7) of case notes discussed factors related to participants’ physical environment. One individual described experiencing violence within their home, which directly impeded their ability to focus on recovery. Others pointed to environmental stressors within their neighborhoods, expressing the need to relocate to safer living environments—specifically those free from the influence of others actively using substances, as notes described, “Currently has been residing in hotels due to the ongoing violence in front of her home, she is looking to move because she is afraid,” (ID 1756, 14th Monthly Meeting, Not in Treatment, 56 Years, African American, Female) and “[Participant] has goals to work again and has recently moved away from a toxic area/home” (ID 1177, 7th Quarterly Meeting, Not in Treatment, 46 Years, African American, Male). Only one participant indicated that living in their new environment reduced the temptation they felt to use substances, “She currently is residing outside of the city and feels good about it, since there is no temptation” (ID 2064, 5th Quarterly Meeting, Not in Treatment, 33 Years, Caucasian, Female).

## Discussion

Study findings shed light on the lived experience of individuals who face numerous challenges to accessing OUD treatment and sustaining recovery following their release from jail. Regarding the primary research question on facilitators of treatment engagement and recovery, both immediate and community social support facilitated participants’ motivation to participate in treatment and interact with supportive social networks. Regarding the secondary research question on barriers to treatment and recovery engagement, case notes identified unemployment, lack of transportation, housing instability, neighborhood violence, negative social influences, poor physical health, and CLS involvement as common barriers. Together, these findings reinforce the importance of structural barriers to OUD treatment participation and reveal directions for enhancing motivational approaches in populations with CLS-involvement.

Barriers experienced by participants in this study align with multiple studies on linking individuals to OUD services during reentry – most commonly to MOUDs [13, 14, 51–53]. These studies have consistently highlighted how unmet SDOH needs during reentry, particularly housing, make it difficult for individuals to prioritize MOUD participation and be retained in services. For example, Kaplowitz and colleagues [14], in a qualitative study of individuals anticipating reentry (*n* = 40), described how transportation and housing barriers had led them to drop-out of methadone services in the past, such as losing their only transportation to the dosing clinic after their car broke down. Multiple qualitative studies describe how competing priorities can take precedent over treatment engagement, and that the stress of not having SDOH-related resources can consume the cognitive capacity needed to focus on this goal [52, 53]. This is not to suggest that all structural barriers must be resolved before psychological change is possible; several studies demonstrate that MI-based interventions can lead to increases in motivation for change and self-efficacy, treatment engagement, and use of coping strategies among participants facing housing, employment, and legal challenges [28, 54–56]. Rather, additional support during the critical period of reentry can help increase motivation to sustain behavioral change. Overall, this study expands the understanding of SDOH factors during reentry beyond MOUD linkage. Participants in this study shared that SDOH factors were also barriers or facilitators of participation in residential and outpatient treatment, and community-based recovery supports.

However, findings reveal other competing priorities that are less commonly described in CLS reentry literature, like caretaking responsibilities and legal documentation. Although the role families play in caring for individuals with OUD is well documented [57], relatively less attention is paid to situations where an individual with OUD is themselves a caretaker – either as a parent or caring for an elderly and/or disabled family member. A study of women using illicit opioids in New York found that mothers were less likely to engage with treatment for fear of involvement with child welfare services and losing custody of their children [58], an experience also documented in the case notes of mothers in RMC. Participants also served as caretakers for elderly family members, emphasizing the need for OUD interventions to provide resources or referral to caretaking services beyond children. Finally, case notes described the barrier of lacking documentation, including insurance and identification documents, that are typically requested during intake processes for treatment services. Interventions can ensure accessibility by integrating protocols for recipients to gain documentation as a part of services.

### Implications for motivational interventions

Consistent with the MI approach employed in the RMC intervention, linkage managers typically build on participant’s identified goals to support their confidence and self-efficacy in taking next steps to achieve these goals. With regard to barriers, structural factors related to unstable housing, employment, or transportation as well as limited resources for caregiving impeded their ability to engage in treatment. Moreover, the perception of overwhelming structural barriers, and repeated prior attempts at treatment, often dampened participants’ motivation to seek treatment [59]. In these instances, linkage managers express empathy and offer to help participants problem-solve how to approach these challenges, again focusing on their self-efficacy and confidence to move forward.

Our study findings suggest the need to acknowledge and account for structural barriers that participants face within the context of a structured intervention designed to link individuals to SUD treatment. Motivational interventions alone may have limited impact on long-term SUD outcomes when participants face structural barriers such as housing instability, poverty, and ongoing CLS involvement. Potential strategies include ensuring motivational interventions are integrated with interdisciplinary care environments (e.g., certified behavioral community health clinics) or community-based settings (e.g., recovery community organizations) that are equipped with staff to help build SDOH-related resources. Alternatively, MI has recently been tested in combination with “resource mobilization,” in which participants are screened for unmet needs during the intervention and linked to housing, transportation, or financial support services, while MI simultaneously targets behavioral change [30].

Maintaining engagement when participants are overwhelmed by structural barriers begins with conveying empathy and honoring autonomy. In many cases it may be clear to the linkage manager that entering treatment *first* will give the individual access to housing and employment options. Resisting the “fixing reflex” [MI 4 term] when these options are refused is critical to maintaining engagement. Within the context of a positive, trusting relationship, linkage managers can meet participants “*where they are at*” by collaborating with participants using concrete, solution-focused steps that respond to participants’ lived realities. For example, linkage managers can help participants create brief, achievable goals that address structural issues (e.g., obtaining identification or contacting a housing hotline), or navigate community resources through visual aid maps. If linkage managers hear ambivalence around pursuing these goals, they can invite the participant to engage in an MI discussion around this new specific action.

### Clinical implications

Case notes frequently described family, mutual aid (i.e., 12-step groups), and faith-based support systems as decisive in motivating and sustaining recovery. Family is noted in prior studies as powerful sources of motivation and change for individuals with substance use issues, but these connections are rarely targeted in intervention [60, 61]. Families can play a critical role in providing help with basic needs, such as monetary aid and access to food and housing [62]. Approaches like Community Reinforcement and Family Training (CRAFT), however, have demonstrated the ability to engage family members actively in treatment planning and recovery support [63, 64]. Recent research has examined how family members who are part of an individual’s “endogenous support system” can be leveraged in re-entry interventions delivered by professionals or paraprofessionals [65].

Equally critical are the implications for the workforce delivering these interventions. Linkage managers, who serve as navigators and motivational partners, often witness first-hand the compounding trauma, systemic neglect, and social inequities their participants face. While they are trained to respect an individual’s autonomy, many describe the emotional toll of navigating participants’ ambivalence in a context where treatment is not readily accessible or responsive. This can generate feelings of “moral injury,” or the psychological distress that arises when professionals are unable to uphold their ethical commitments due to institutional constraints [66, 67]. In frontline roles, the result is often compassion fatigue, vicarious trauma, and frustration with systems that repeatedly fail their participants [68–70]. These challenges demand more than individual self-care. Strengthening linkage manager resilience requires institutional supports such as regular reflective supervision, peer-led debriefing groups, and sessions with trauma specialists [71]. Long-term success of motivational interventions like RMC thus depends not only on addressing participant barriers, but also on supporting the well-being of those tasked with helping participants navigate them.

A distinguishing feature of the RMC intervention is its singular focus on helping individuals to access SUD treatment and to sustain treatment engagement and recovery [37]. This focus differentiates linkage managers from the broader function of case managers, who often serve as service brokers across various service providers. The RMC model stresses the importance of maintaining this focus on treatment engagement as a bridge to the broader range of options that are possible when individuals experience the benefits of recovery, such as improved health, cognitive capacity, family functioning, and well-being. Yet linkage managers frequently voiced frustrations about their inability to address the broader set of participant needs as well as the structural constraints they operate within. Incorporating interdisciplinary case conferences or cultivating relationships with key stakeholders in other service systems can enhance the capacity of linkage managers at the same time they concentrate on helping individuals access SUD treatment and recovery support services.

### Study limitations

Study findings are most applicable to similar populations, i.e., older individuals, largely African American, with CLS involvement, mainly from a large urban area. As such, these findings should not be assumed to generalize to individuals with CLS involvement in non-urban regions, or to populations with different racial/ethnic compositions. Importantly, this study relied on case notes written by linkage managers rather than direct participant interviews. Findings therefore reflect linkage managers’ interpretations and documentation of participants’ experiences, rather than participants’ own words or perspectives. Case notes used in the analysis likely reflected the perceptions of individual linkage managers and were filtered by their personal biases, beliefs, or perceptions of salient factors. There were no quality control procedures for case notes, although a study investigator reviewed them weekly and provided feedback or queried for additional information. Additionally, the linkage managers met regularly with the MI trainer who provided ongoing coaching and feedback on MI strategies for engaging study participants. Although coded data were aggregated by participant to reduce the influence of individuals whose notes appeared more frequently in the analytic sample, some participants from the parent trial may still be represented more than others due to sampling structure. Finally, member reflection was conducted with linkage staff rather than participants themselves. Because this enhanced contextual interpretation of RMC practice is from the perspective of linkage managers, it does not constitute participant validation of findings and may limit the degree to which results reflect participants’ intended meanings.

## Conclusions

The study findings provide documentation of the range and complexity of social determinants of health that individuals with OUD recently released from jail describe as supporting or impeding their pathways to treatment and recovery. Substance use and behavioral health counselors, peer recovery workers, and case managers are likely to encounter these issues when working with similar populations that face economic, health, and environmental challenges. These findings can inform training protocols for interventions targeting this population, including ways to bolster motivational interviewing and linkage interventions to address the extensive and challenging issues that hinder an individual’s ability to engage in treatment or sustain recovery.

## Data Availability

The dataset analyzed during the current study is available from the corresponding author on reasonable request.

## Abbreviations

SDOH: Social Determinants of Health
OUD: Opioid use disorder
MOUD: Medications for opioid use disorder
SUD: Substance use disorder
CLS: Criminal legal system
MI: Motivational interviewing
JCOIN: Justice Community Opioid Innovation Network
RMC: Recovery Management Checkups
CRAFT: Community Reinforcement and Family Training

## References

[1] Lin C, Cousins SJ, Zhu Y, Clingan SE, Mooney LJ, Kan E, et al. A scoping review of social determinants of health’s impact on substance use disorders over the life course. J Subst Use Addict Treat. 2024;166:209484. 10.1016/j.josat.2024.209484. 39153733 10.1016/j.josat.2024.209484 PMC11418584

[2] Marmot M, Wilkinson R. Social determinants of health. OUP Oxford. 2005;465.

[3] Navarro V. What we mean by social determinants of health. Glob Health Promot. 2009;16(1):05–16. 10.1177/1757975908100746. 10.1177/1757975908100746 19276329

[4] Spooner C, Hetherington K. Social determinants of drug use [Internet]. National drug & alcohol research center. 2005 [cited 2025 Jul 2]. Report: NDARC Technical Report. Available from: https://www.unsw.edu.au/research/ndarc/resources/social-determinants-of-drug-use.

[5] Gaeta Gazzola M, Carmichael ID, Christian NJ, Zheng X, Madden LM, Barry DT. A National Study of Homelessness, Social Determinants of Health, and Treatment Engagement Among Outpatient Medication for Opioid Use Disorder-Seeking Individuals in the United States. Substance abuse. 2023;44(1–2):62–72. 10.1177/08897077231167291. 37226909 10.1177/08897077231167291

[6] Krawczyk N, Williams AR, Saloner B, Cerdá M. Who stays in medication treatment for opioid use disorder? A national study of outpatient specialty treatment settings. J Subst Abuse Treat. 2021;126:108329. 10.1016/j.jsat.2021.108329. 34116820 10.1016/j.jsat.2021.108329 PMC8197774

[7] Sahker E, Ali SR, Arndt S. Employment recovery capital in the treatment of substance use disorders: Six-month follow-up observations. Drug Alcohol Depend. 2019;205:107624. 10.1016/j.drugalcdep.2019.107624. PubMed PMID: 31645013. 31645013 10.1016/j.drugalcdep.2019.107624

[8] Zhang W, Wu H. The Relationship of Socioeconomic Factors and Substance Abuse Treatment Dropout. Healthcare. 2025;13(4):4. 10.3390/healthcare13040369. 10.3390/healthcare13040369 PMC11855641 39997244

[9] Eddie D, Prindle J, Somodi P, Gerstmann I, Dilkina B, Saba SK, et al. Exploring predictors of substance use disorder treatment engagement with machine learning: The impact of social determinants of health in the therapeutic landscape. J Subst Use Addict Treat. 2024;164:209435. 10.1016/j.josat.2024.209435. 38852819 10.1016/j.josat.2024.209435 PMC11300147

[10] Justice center at the council of state governments. The role of probation and parole in making housing a priority for people with behavioral health needs [Internet]. 2021. (Field Notes | Behavioral Health). Report. Available from: https://csgjusticecenter.org/wp-content/uploads/2021/03/Field-Notes_The-Role-of-Probation-and-Parole-in-Housing.pdf.

[11] Medicaid CHIP, Payment, and access commission. Access in brief: health care needs of adults involved with the criminal justice system [Internet]. Washington, DC: MACPAC. 2021. (Advising Congress on MedicaidCHIP Policy). Report. Available from: https://www.macpac.gov/wp-content/uploads/2021/08/Access-in-Brief-Health-Care-Needs-of-Adults-Involved-with-the-Criminal-Justice-System.pdf.

[12] Widra E, Jones A. Mortality, health, and poverty: the unmet needs of people on probation and parole [Internet]. Prison policy initative. 2023 [cited 2025 Jul 16]. Report. Available from: https://www.prisonpolicy.org/blog/2023/04/03/nsduh_probation_parole/.

[13] Owens MD, Chen JA, Simpson TL, Timko C, Williams EC. Barriers to addiction treatment among formerly incarcerated adults with substance use disorders. Addict Sci Clin Pract. 2018;13:19. 10.1186/s13722-018-0120-6. PubMed PMID: 30126452; PubMed Central PMCID: PMC6102909. 30126452 10.1186/s13722-018-0120-6 PMC6102909

[14] Kaplowitz E, Truong A, Macmadu A, Berk J, Martin H, Burke C, et al. Anticipated Barriers to Sustained Engagement in Treatment With Medications for Opioid Use Disorder After Release From Incarceration. J Addict Med. 2023;17(1):54–9. PubMed PMID: 35916404; PubMed Central PMCID: PMC9892350. 35916404 10.1097/ADM.0000000000001029 PMC9892350

[15] Chladek JS, Chui MA. Access to medications for opioid use disorder for formerly incarcerated individuals during community reentry: a mini narrative review. Front Public Health. 2024;12:1377193. 10.3389/fpubh.2024.1377193. PubMed PMID: 38803812; PubMed Central PMCID: PMC11128549. 38803812 10.3389/fpubh.2024.1377193 PMC11128549

[16] Martin RA, Alexander-Scott N, Berk J, Carpenter RW, Kang A, Hoadley A, et al. Post-incarceration outcomes of a comprehensive statewide correctional MOUD program: a retrospective cohort study. Lancet Reg Health Am. 2023;18:100419. 10.1016/j.lana.2022.100419. PubMed PMID: 36844014; PubMed Central PMCID: PMC9950664. 36844014 10.1016/j.lana.2022.100419 PMC9950664

[17] DeRoche C. Changing policy responses to drug use must be shaped by a new perspective on addiction [Internet]. Chicago, IL: Safety + Justice challenge at the MacArthur foundation. 2020 [cited 2025 Jul 28]. Report. Available from: https://safetyandjusticechallenge.org/blog/changing-policy-responses-drug-use-must-shaped-new-perspective-addiction/.

[18] Polcin DL. Reexamining Confrontation and Motivational Interviewing. Addictive Disorders. Their Treat. 2006;5(4):201. 10.1097/01.adt.0000205048.44129.6a.

[19] White W, Miller W. The use of confrontation in addiction treatment history, science, and time for change a history of confrontational therapies. Counselor. 2007;8.

[20] Miller WR, Rollnick S. Motivational interviewing: helping people change and grow. Guilford; 2023;354.

[21] Rollnick S, Miller WR, Butler CC. Motivational interviewing in health care: Helping patients change behavior. New York, NY, US: Guilford Press. 2008;210.

[22] Schwenker R, Dietrich CE, Hirpa S, Nothacker M, Smedslund G, Frese T, et al. Motivational interviewing for substance use reduction. Cochrane Database Syst Rev. 2023;12(12):CD008063. 10.1002/14651858.CD008063.pub3. PubMed PMID: 38084817; PubMed Central PMCID: PMC10714668. 38084817 10.1002/14651858.CD008063.pub3 PMC10714668

[23] US. Department of health and human services. TIP 35: Enhancing motivation for change in substance use disorder treatment (Updated 2019). Lulu.com. 2019;208. 34106565

[24] Newbury-Birch D, Ferguson J, Connor N, Divers A, Waller G. A Rapid systematic review of worldwide alcohol use disorders and brief alcohol interventions in the criminal justice system. Front Psychiatry. 2022;13. 10.3389/fpsyt.2022.900186. 10.3389/fpsyt.2022.900186 PMC9301009 35873244

[25] Pennsylvania juvenile court judges commission. Implementation survey: Juvenile justice system enhancement strategy [Internet]. Harrisburg, PA: Pennsylvania Juvenile Court Judges Commission. 2019 [cited 2026 Feb 19]. Report. Available from: https://www.pa.gov/content/dam/copapwp-pagov/en/jcjc/documents/publications/jjses/2019%20jjses%20implementation%20survey%20results.pdf.

[26] Walker A, Fixsen D, Van Dyke M, Imm P, Williams A, Hyde J, et al. Motivational Interviewing for Community Corrections: Expanding a Relationship-based Approach with Exemplar Implementation. Fed Probat. 2020;84(2):35–43.

[27] Iarussi M, Powers DF. Outcomes of motivational interviewing training with probation and parole officers: Findings and lessons learned. Federal Probation Journal [Internet]. 2018 Dec 1;82(3) [cited 2026 Feb 19]. Available from: https://www.uscourts.gov/about-federal-courts/probation-and-pretrial-services/federal-probation-journal/2018/12/outcomes-motivational-interviewing-training-probation-and-parole-officers-findings-and-lessons.

[28] Pinto e Silva T, Gouveia C, Santirso FA, Cunha O, Caridade S. Effectiveness of Motivational Interviewing with Justice-involved People: A Systematic Review and Meta-analysis. Psychosoc Interv. 2025;34(2):89–102. 10.5093/pi2025a. 8 PubMed PMID: 40405915; PubMed Central PMCID: PMC12097221. 40405915 10.5093/pi2025a8 PMC12097221

[29] Owens MD, McCrady BS. A Pilot Study of a Brief Motivational Intervention for Incarcerated Drinkers. J Subst Abuse Treat. 2016;68:1–10. 10.1016/j.jsat.2016.05.005. PubMed PMID: 27431041; PubMed Central PMCID: PMC4955738. 27431041 10.1016/j.jsat.2016.05.005 PMC4955738

[30] Laroche HH, Park-Mroch J, O’Shea A, Rice S, Cintron Y, Engebretsen B. Resource mobilization combined with motivational interviewing to promote healthy behaviors and healthy weight in low-income families: An intervention feasibility study. SAGE Open Med. 2022;10:20503121221102706. 10. 1177/20503121221102706 PubMed PMID: 35707344; PubMed Central PMCID: PMC9189556. 35707344 10.1177/20503121221102706 PMC9189556

[31] Oyefusi V, South-Paul. Addressing healthcare access and disparities using motivational interviewing. In: Douaihy A, Kelly TM, Gold MA, editors. Motivational interviewing: a guide for medical trainees. Oxford University Press. 2023.

[32] Alcántara C, Diaz SV, Cosenzo LG, Loucks EB, Penedo FJ, Williams NJ. Social Determinants as Moderators of the Effectiveness of Health Behaviour Change Interventions: Scientific Gaps and Opportunities. Health Psychol Rev. 2020;14(1):132–44. PubMed PMID: 31957557; PubMed Central PMCID: PMC11600431. 31957557 10.1080/17437199.2020.1718527 PMC11600431

[33] Sorbello L, Eccleston L, Ward T, Jones R. Treatment needs of female offenders: a review. Australian Psychol. 2002;37(3):198–205. 10.1080/00050060210001706876.

[34] de Castro Rodrigues A, Andrade J, Gonçalves RA, Cruz AR, Cunha O. or Out: Justice-Involved Women Characterization and Their Perceptions about Penal Sanctions. Women Criminal Justice. 2025;35(4):248–65. 10.1080/08974454.2022.2126743.

[35] Dennis M, Scott CK, Funk R. An experimental evaluation of recovery management checkups (RMC) for people with chronic substance use disorders. Evaluation and program planning. Longitud Evaluat Subst Abuse Treat. 2003;26(3):339–52. 10.1016/S0149-71890300037-5. 10.1016/S0149-7189(03)00037-5 PMC6054319 30034059

[36] Dennis ML, Scott CK. Four-year outcomes from the Early Re-Intervention (ERI) experiment using Recovery Management Checkups (RMCs). Drug Alcohol Depend. 2012;121(1):10–7. 10.1016/j.drugalcdep.2011.07.026. 21903347 10.1016/j.drugalcdep.2011.07.026 PMC3277866

[37] Scott CK, Dennis ML, Foss MA. Utilizing Recovery Management Checkups to shorten the cycle of relapse, treatment reentry, and recovery. Drug Alcohol Depend. 2005;78(3):325–38. 10.1016/j.drugalcdep.2004.12. PubMed PMID: 15893164; PubMed Central PMCID: PMC5933845. 15893164 10.1016/j.drugalcdep.2004.12.005 PMC5933845

[38] Scott CK, Grella CE, Nicholson L, Dennis ML. Opioid recovery initiation: Pilot test of a peer outreach and modified Recovery Management Checkup intervention for out-of-treatment opioid users. J Subst Abuse Treat. 2018;86:30–5. 10.1016/j.jsat.2017.12.007. 29415848 10.1016/j.jsat.2017.12.007 PMC5808598

[39] Scott CK, Dennis ML, Grella CE, Watson DP, Davis JP, Hart MK. Using recovery management checkups for primary care to improve linkage to alcohol and other drug use treatment: a randomized controlled trial three month findings. Addiction. 2023;118(3):520–32. 10.1111/add.16064. PubMed PMID: 36208061; PubMed Central PMCID: PMC10015976. 36208061 10.1111/add.16064 PMC10015976

[40] Scott CK, Dennis ML. Results from two randomized clinical trials evaluating the impact of quarterly recovery management checkups with adult chronic substance users. Addiction. 2009;104(6):959–71. 10.1111/j.1360-0443.2009.02525.x. 19344441 10.1111/j.1360-0443.2009.02525.x PMC2695999

[41] Scott CK, Dennis ML. The first 90 days following release from jail: Findings from the Recovery Management Checkups for Women Offenders (RMCWO) experiment. Drug Alcohol Depend. 2012;125(1):110–8. 10.1016/j.drugalcdep.2012.03.025. 22542465 10.1016/j.drugalcdep.2012.03.025 PMC3419296

[42] Watson DP, Singh R, Taylor L, Dennis ML, Grella CE, Johnstone C, et al. Exploring the feasibility of Recovery Management Checkups for Primary Care in a Federally Qualified Health Center. Front Public Health. 2024;12:1443409. 10.3389/fpubh.2024.1443409. PubMed PMID: 39588163; PubMed Central PMCID: PMC11586366. 39588163 10.3389/fpubh.2024.1443409 PMC11586366

[43] Keloth VK, Selek S, Chen Q, Gilman C, Fu S, Dang Y, et al. Social determinants of health extraction from clinical notes across institutions using large language models. npj Digit Med. 2025;8(1):287. 10.1038/s41746-025-01645-8. 40379919 10.1038/s41746-025-01645-8 PMC12084648

[44] Kepper MM, Walsh-Bailey C, Prusaczyk B, Zhao M, Herrick C, Foraker R. The adoption of social determinants of health documentation in clinical settings. Health Serv Res. 2023;58(1):67–77. 10.1111/1475-6773.14039. PubMed PMID: 35862115; PubMed Central PMCID: PMC9836948. 35862115 10.1111/1475-6773.14039 PMC9836948

[45] Scott CK, Dennis ML, Grella CE, Watson DP. Improving Retention across the OUD Service Cascade upon Reentry from Jail using Recovery Management Checkups Adaptive (RMC-A) Experiment. J Subst Abuse Treat. 2021;128:108245. 10.1016/j.jsat.2020.108245. PubMed PMID: 33461829; PubMed Central PMCID: PMC8192586. 33461829 10.1016/j.jsat.2020.108245 PMC8192586

[46] Assarroudi A, Heshmati Nabavi F, Armat MR, Ebadi A, Vaismoradi M. Directed qualitative content analysis: the description and elaboration of its underpinning methods and data analysis process. J Res Nurs. 2018;23(1):42–55. PubMed PMID: 34394406; PubMed Central PMCID: PMC7932246. 34394406 10.1177/1744987117741667 PMC7932246

[47] Gale NK, Heath G, Cameron E, Rashid S, Redwood S. Using the framework method for the analysis of qualitative data in multi-disciplinary health research. BMC Med Res Methodol. 2013;13:117. 10.1186/1471-2288-13-117. PubMed PMID: 24047204; PubMed Central PMCID: PMC3848812. 24047204 10.1186/1471-2288-13-117 PMC3848812

[48] Ayres L, Kavanaugh K, Knafl KA. Within-case and across-case approaches to qualitative data analysis. Qual Health Res. 2003;13(6):871–83. PubMed PMID: 12891720. 12891720 10.1177/1049732303013006008

[49] Birt L, Scott S, Cavers D, Campbell C, Walter F. Member Checking: A Tool to Enhance Trustworthiness or Merely a Nod to Validation? Qual Health Res. 2016;26(13):1802–11. PubMed PMID: 27340178. 27340178 10.1177/1049732316654870

[50] Candela A. Exploring the function of member checking. The qualitative report. 2019 Mar 24. 10.46743/2160-3715/2019.3726.

[51] Hoffman KA, Thompson E, Gaeta Gazzola M, Oberleitner LMS, Eller A, Madden LM, et al. Just fighting for my life to stay alive: a qualitative investigation of barriers and facilitators to community re-entry among people with opioid use disorder and incarceration histories. Addict Sci Clin Pract. 2023;18(1):16. 10.1186/s13722-023-00377-y. 36944998 10.1186/s13722-023-00377-y PMC10031976

[52] Fox AD, Maradiaga J, Weiss L, Sanchez J, Starrels JL, Cunningham CO. Release from incarceration, relapse to opioid use and the potential for buprenorphine maintenance treatment: a qualitative study of the perceptions of former inmates with opioid use disorder. Addict Sci Clin Pract. 2015;10(1):2. 10.1186/s13722-014-0023-0. PubMed PMID: 25592182; PubMed Central PMCID: PMC4410477. 25592182 10.1186/s13722-014-0023-0 PMC4410477

[53] Howard H, Skinner-Osei P, Mitchell C, Cadavid E, Hulick J. Now I Have My Own Key: The Impact of Housing Stability on Recovery and Recidivism Reduction Using a Recovery Capital Framework. Urban Social Work. 2023;7(2):116–35. 10.1891/USW-2023-0004.

[54] Britt E, Soleymani S, Wallace-Bell M, Garland A. Motivational interviewing for employment: An exploration of practitioner skill and client change talk. J Employ Couns. 2023;60(1):42–59. 10.1002/joec.12198.

[55] Kennedy DP, Osilla KC, Hunter SB, Golinelli D, Maksabedian Hernandez E, Tucker JS. A pilot test of a motivational interviewing social network intervention to reduce substance use among housing first residents. J Subst Abuse Treat. 2018;86:36–44. 10.1016/j.jsat.2017.12.005. PubMed PMID: 29415849; PubMed Central PMCID: PMC5808606. 29415849 10.1016/j.jsat.2017.12.005 PMC5808606

[56] Mera S. The use of motivational interviewing with chronically unhoused veterans. Clin Soc Work J. 2025 Jan 14. 10.1007/s10615-025-00984-z.

[57] Maina G, Ogenchuk M, Phaneuf T, Kwame A. I can’t live like that: the experience of caregiver stress of caring for a relative with substance use disorder. Subst Abuse Treat Prev Policy. 2021;16(1):11. 10.1186/s13011-021-00344-3. 33446208 10.1186/s13011-021-00344-3 PMC7809821

[58] Scheidell JD, Hoff L, Khan MR, Bennett AS, Elliott L. Parenting and childcare responsibilities, harm reduction service engagement, and opioid overdose among women and men who use illicit opioids in New York City. Drug Alcohol Depend Rep. 2022;3:100054. 10.1016/j.dadr.2022.100054. 35757568 10.1016/j.dadr.2022.100054 PMC9224239

[59] Farhoudian A, Razaghi E, Hooshyari Z, Noroozi A, Pilevari A, Mokri A, et al. Barriers and Facilitators to Substance Use Disorder Treatment: An Overview of Systematic Reviews. Subst Abuse. 2022;16:11782218221118462. 10.1177/11782218221118462. 36062252 10.1177/11782218221118462 PMC9434658

[60] Hogue A, Becker SJ, Wenzel K, Henderson CE, Bobek M, Levy S, et al. Family Involvement in Treatment and Recovery for Substance Use Disorders among Transition-Age Youth: Research Bedrocks and Opportunities. J Subst Abuse Treat. 2021;129:108402. 10.1016/j.jsat.2021.108402. PubMed PMID: 34080559; PubMed Central PMCID: PMC8380649. 34080559 10.1016/j.jsat.2021.108402 PMC8380649

[61] Kourgiantakis T, Ashcroft R. Family-focused practices in addictions: a scoping review protocol. BMJ Open. 2018;8(1):e019433. 10.1136. /bmjopen-2017-019433 PubMed PMID: 29331973; PubMed Central PMCID: PMC5781095. 29331973 10.1136/bmjopen-2017-019433 PMC5781095

[62] Mowen TJ, Stansfield R, Boman JH. Family Matters: Moving Beyond If Family Support Matters to Why Family Support Matters during Reentry from Prison. J Res Crime Delinq. 2019;56(4):483–523. PubMed PMID: 32382195; PubMed Central PMCID: PMC7205225. 32382195 10.1177/0022427818820902 PMC7205225

[63] Bischof G, Iwen J, Freyer-Adam J, Rumpf HJ. Efficacy of the Community Reinforcement and Family Training for concerned significant others of treatment-refusing individuals with alcohol dependence: A randomized controlled trial. Drug Alcohol Depend. 2016;163:179–85. 10.1016/j.drugalcdep.2016.04.015. 27141840 10.1016/j.drugalcdep.2016.04.015

[64] Kirby KC, Versek B, Kerwin ME, Meyers K, Benishek LA, Bresani E, et al. Developing Community Reinforcement and Family Training (CRAFT) for Parents of Treatment-Resistant Adolescents. J Child Adolesc Subst Abuse. 2015;24(3):155–65. PubMed PMID: 25883523; PubMed Central PMCID: PMC4394369. 25883523 10.1080/1067828X.2013.777379 PMC4394369

[65] Schneider JA, Smith LR, Bouris AM, Oser C, Pho MT, Boodram B, et al. A taxonomy of social support interventions for People experiencing a syndemic of substance use disorder, criminal legal involvement, and downstream Health sequelae. SSM Ment Health. 2025;7:100428. 10.1016/j.ssmmh.2025.100428. PubMed PMID: 40677743; PubMed Central PMCID: PMC12269891. 40677743 10.1016/j.ssmmh.2025.100428 PMC12269891

[66] Čartolovni A, Stolt M, Scott PA, Suhonen R. Moral injury in healthcare professionals: A scoping review and discussion. Nurs Ethics. 2021;28(5):590–602. PubMed PMID: 33427020; PubMed Central PMCID: PMC8366182. 33427020 10.1177/0969733020966776 PMC8366182

[67] Harper S, Karypidou A. Moral suffering in frontline social care workers: a study of moral injury and moral distress. Eur J Mental Health. 2024;(e0021):e0021.

[68] Alavi S, Nishar S, Morales A, Vanjani R, Guy A, Soske J. We need to get paid for our value: Work-place experiences and role definitions of peer recovery specialists/community health workers. Alcohol Treat Q. 2024;42(1):95–114. PubMed PMID: 38352063; PubMed Central PMCID: PMC10861181. 38352063 10.1080/07347324.2023.2272797 PMC10861181

[69] Fahy A. The Unbearable Fatigue of Compassion: Notes from a Substance Abuse Counselor Who Dreams of Working at Starbuck’s. Clin Soc Work J. 2007;35(3):199–205. 10.1007/s10615-007-0094-4.

[70] Guy AA, Nishar S, Alavi S, Morales A, Vanjani R, Soske J. Carrying the Weight of a Broken System: Community Health Worker and Peer Recovery Specialist roles transformed. Prog Community Health Partnersh. 2025;19(1):129–35. 10.1353/cpr.2025.a956604. PubMed PMID: 40223635; PubMed Central PMCID: PMC11999244. 40223635 10.1353/cpr.2025.a956604 PMC11999244

[71] Jones CT, Branco SF. Trauma-Informed Supervision: Clinical Supervision of Substance Use Disorder Counselors. J Addictions Offender Couns. 2020;41(1):2–17. 10.1002/jaoc.12072.

